# TupA: A Tungstate Binding Protein in the Periplasm of *Desulfovibrio alaskensis* G20

**DOI:** 10.3390/ijms150711783

**Published:** 2014-07-02

**Authors:** Ana Rita Otrelo-Cardoso, Rashmi R. Nair, Márcia A. S. Correia, Maria G. Rivas, Teresa Santos-Silva

**Affiliations:** 1 Rede de Química e Tecnologia/Centro de Química Fina e Biotecnologia (REQUIMTE/CQFB), Department of Chemistry, Faculdade de Ciências e Tecnologia, Universidade Nova de Lisboa, Caparica 2829-516, Portugal; E-Mails: a.cardoso@campus.fct.unl.pt (A.R.O.-C.); r.nair@fct.unl.pt (R.R.N.); marcia.correia@fct.unl.pt (M.A.S.C.); 2 Department of Physics, Facultad de Bioquímica y Ciencias Biológicas, Universidad Nacional del Litoral, Santa Fe 3000, Argentina

**Keywords:** TupA, tungstate, metal transport, *Desulfovibrio*, sulfate reducing bacteria, protein-ligand interaction, isothermal titration calorimetry, X-ray crystallography

## Abstract

The TupABC system is involved in the cellular uptake of tungsten and belongs to the ABC (ATP binding cassette)-type transporter systems. The TupA component is a periplasmic protein that binds tungstate anions, which are then transported through the membrane by the TupB component using ATP hydrolysis as the energy source (the reaction catalyzed by the ModC component). We report the heterologous expression, purification, determination of affinity binding constants and crystallization of the *Desulfovibrio alaskensis* G20 TupA. The *tupA* gene (locus tag Dde_0234) was cloned in the pET46 Enterokinase/Ligation-Independent Cloning (LIC) expression vector, and the construct was used to transform BL21 (DE3) cells. TupA expression and purification were optimized to a final yield of 10 mg of soluble pure protein per liter of culture medium. Native polyacrylamide gel electrophoresis was carried out showing that TupA binds both tungstate and molybdate ions and has no significant interaction with sulfate, phosphate or perchlorate. Quantitative analysis of metal binding by isothermal titration calorimetry was in agreement with these results, but in addition, shows that TupA has higher affinity to tungstate than molybdate. The protein crystallizes in the presence of 30% (*w*/*v*) polyethylene glycol 3350 using the hanging-drop vapor diffusion method. The crystals diffract X-rays beyond 1.4 Å resolution and belong to the P2_1_ space group, with cell parameters *a* = 52.25 Å, *b* = 42.50 Å, *c* = 54.71 Å, β = 95.43°. A molecular replacement solution was found, and the structure is currently under refinement.

## 1. Introduction

Molybdenum and tungsten are trace elements used by almost all forms of life. Since Mo and W atoms share several similar chemical characteristics, biological systems have to develop strategies to differentiate one metal from the other and to avoid the incorrect metal insertion in the active site of enzymes [1,2]. These metals enter the cell as soluble oxoanions, MoO_4_^2−^ and WO_4_^2−^, through specific ATP-binding cassette (ABC) transporter systems. In prokaryotes, these transport systems are divided into three different families: Mod, Wtp and Tup. All of these systems are composed of a periplasmic protein (component A), a transmembrane pore forming protein (component B) and a cytoplasmic protein (component C), which hydrolyzes ATP to generate the energy necessary to transport the oxoanion into the cell cytoplasm [2,3,4,5]. The genes encoding the three components are organized in an operon (*mod*/*wtpABC*) or gene cluster (*tupABC*) regulated by a transcription factor known as ModE in the case of the ModABC operon. Under an excess of molybdate, ModE binds molybdate ions, suffers conformational changes and dimerizes. This metal-protein complex binds to a specific DNA sequence (located upstream of the *modABC* operon) and downregulates the expression of proteins involved in molybdenum uptake [4,6,7,8].

Under oxoanion starvation, the component A binds molybdate or tungstate and interacts with the component B to actively transport molybdate or tungstate from the periplasm to the cytoplasm [4]. Therefore, the Mod/Wtp/TupABC transport system and, more specifically, the component A should constitute the first selection gate from which cells should differentiate between Mo and W. The basis for this selectivity is currently unknown. The periplasmic component of the Mod/Tup/WtpABC system differs not only in the primary sequence, but also in the metal affinity and coordination chemistry of the molybdate/tungstate [2,9,10,11,12,13,14,15,16]. Crystal structures of ModA have already been solved, showing a tetrahedral coordination with five conserved amino acids located at a H-bond donating distance from the oxygen atoms of the oxoanions [17,18,19]. Different from ModA, the tungstate binding protein, WtpA, binds tungstate in a distorted octahedral conformation with two carboxylate oxygens from conserved glutamate (Glu218) and aspartate (Asp160) residues (*Pyrococcus furiosus* (*Pf*) numbering), with several examples in the literature [20]. The oxoanion coordination in TupA protein has not yet been reported, but it is known that the TTTS amino acid sequence at the *N*-terminal amino acid sequence is a signature of this type of tungstate transporters. In this motif, the Thr9 and Ser11 (*Geobacter sulfurreducens* (*Gs*) numbering) are predicted to be interacting with the oxoanion through hydrogen bonds. In addition, a conserved threonine in the *C*-terminal domain, Thr124, is postulated to coordinate the oxoanion through hydrogen bonds [2]. The crystal structure of *Gs* TupA has been deposited in the Protein Data Bank (PDB code 3LR1) with a W^6+^ ion close to the TTTS motif. The binding mode of the ion is not clear and needs to be further scrutinized.

*Desulfovibrio alaskensis* G20 (*Da*G20) is a sulfate reducing bacterium (SRB) that obtains energy from sulfate reduction and produces sulfide, a highly toxic and corrosive metabolite [21]. SRBs are the main cause responsible for a phenomenon known as microbiologically-influenced corrosion (MIC), with very relevant economic consequences in several industries, including the chemical, paper, power, marine and petroleum industry [22,23,24]. Molybdate can be used to control the SRB growth mainly by the inhibition of ATP-sulfurylase, a key enzyme in sulfate activation [25,26,27]. In addition, we have observed that high molybdate concentration in cultures of *Da*G20 affect the expression of proteins involved in energy metabolism, ion transport, cell cycle, amino acid, purines, pyrimidines, nucleosides and nucleotides biosynthesis and other cellular mechanisms. Regarding the proteins involved in ion transport, we found that not only the periplasmic protein involved in molybdate transport (ModA), but also the protein involved in tungstate transport (TupA) are downregulated under these stress conditions [28].

**Figure 1:**
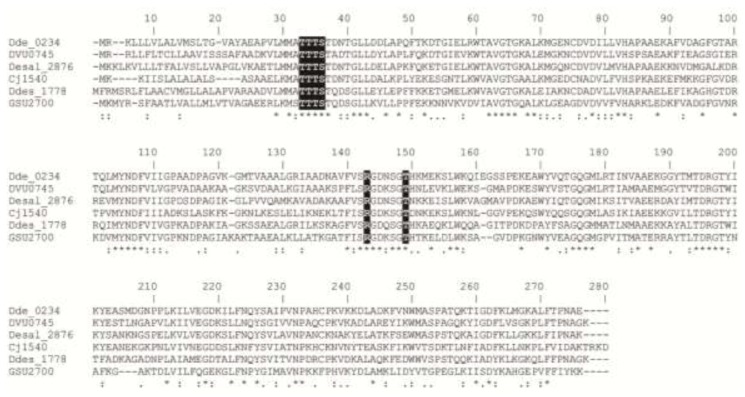
Multiple sequence alignment of TupA proteins performed with ClustalW [29]. Dde_0234, *Desulfovibrio alaskensis* G20; DVU0745, *Desulfovibrio vulgaris* Hildenborough; Dde_2876, *Desulfovibrio salexigens*; Cj1540; *Campylobacter jejuni* strain NCTC 11168; Dde_1778, *Desulfovibrio desulfuricans* ATCC 27774; GSU2700, *Geobacter sulfurreducens*. Residues putatively involved in the coordination of tungstate are highlighted in black. Symbols: (*) identity, (:) strongly similar and (.) weakly similar.

Despite the presence of several relevant Mo- and W-containing enzymes in the *Desulfovibrio* metabolism, there are no reports about molybdate/tungstate transport systems in this organism. Genome analysis shows that it codifies both molybdate and tungstate transporters. The tungstate transport system corresponds to the Tup kind of transporters. Analysis of the primary sequence of the *Da*G20 TupA contains all of the conserved residues putatively involved in the oxoanion coordination [1] (Figure 1).

Here, we report the expression, purification, determination of affinity binding constants and crystallization of the *Da*G20 TupA protein. The high resolution structure (up to 1.4 Å resolution) will provide useful information about the coordination geometry of the oxoanion to the protein. In addition, the expression system and purification protocol described are useful to construct mutants that will make a relevant contribution to the knowledge of the selectivity mechanisms that allow the cell to differentiate between Mo and W.

## 2. Results and Discussion

### 2.1. Cloning of tupA Gene and Purification of TupA Protein

**Figure 2:**
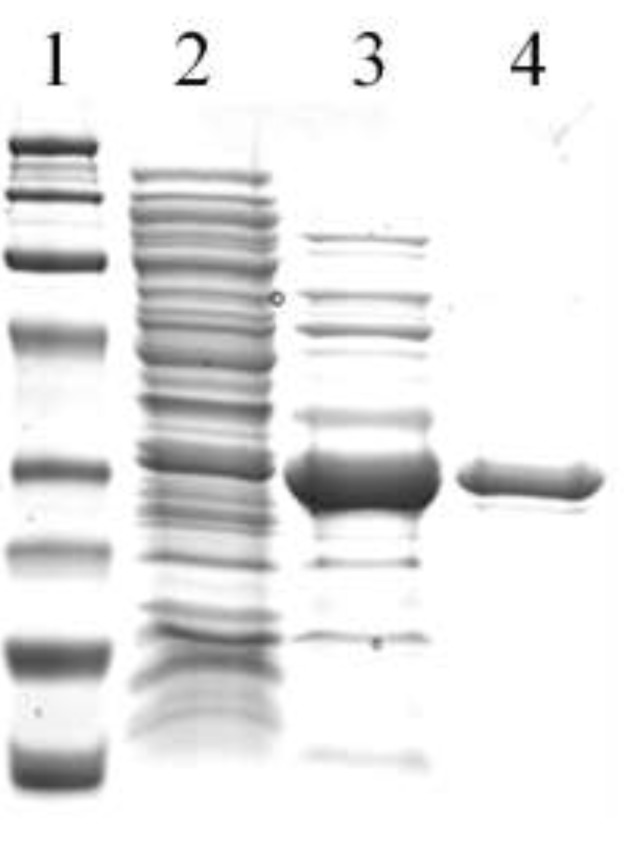
SDS-PAGE stained with Coomassie blue of (**1**) molecular weight markers (Bio-Rad; from top: 100, 75, 50, 37, 25, 20, 15 and 10 kDa); (**2**) the soluble protein fraction; (**3**) the TupA fraction after anionic exchange chromatography; and (**4**) the TupA fraction after molecular exclusion chromatography (approximately 15 µg of pure protein).

The *tupA* gene (Dde_0234) was cloned into the pET-46 Ek/LIC vector using the Ek-LIC cloning system (Novagen, Darmstadt, Germany), and the protein was expressed in BL21 (DE3) cells. The expression level of TupA and the ratio TupA/contaminants were evaluated by SDS-PAGE at different induction times (3 h, 5 h and overnight), and 3 h of induction were considered the optimum condition for TupA production in BL21 (DE3) cells. SDS-PAGE showed that TupA is present in both the soluble and insoluble fraction (data not shown). Since the amount of TupA in the soluble fraction was considered enough to perform the studies here described, we proceed to isolate the protein from this fraction. As explained in the Experimental Section, TupA purification protocol includes two steps, an anionic exchange and a size exclusion chromatography. TupA elutes from the anionic exchange resin at approximately 200 mM Tris-HCl (pH 7.6), which is in agreement with the isoelectric point calculated for the protein (pI 5.69, ProtParam tool [30]). The degree of purity after each purification step was evaluated by SDS-PAGE (Figure 2). According to the protein sequence, the molecular weight of the recombinant protein should be approximately 29 kDa. The purification yield was calculated to be approximately 10 mg of soluble protein per liter of cell culture.

### 2.2. UV-Visible Spectrum and Protein Sequence

**Figure 3:**
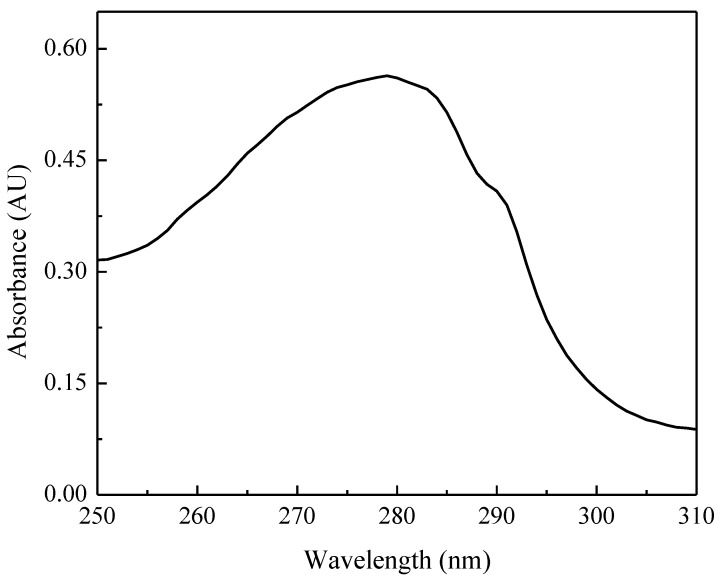
UV-visible spectrum of as isolated TupA protein (0.020 µM protein in 50 mM Tris-HCl pH 7.6).

The UV-visible spectrum of the as-isolated TupA protein is shown in Figure 3. The maximum observed at 280 nm is due to the six Tyr residues present in the primary structure, whereas the shoulder at 288 nm is probably derived from the four Trp residues (Figure 1, Dde_0234).

The extinction coefficient of TupA at 280 nm (29,700 ± 700 M^−1^·cm^−1^) was found to be in good agreement with that deduced from the amino acid sequence of the pure protein (30,440 M^−1^·cm^−1^).

Multiple sequence alignment of TupA proteins shows that the *Da*G20 TupA contains the TTTS motif at the *N*-terminal region, which is the typical signature of this kind of tungstate transporter. The amino acids suggested to form hydrogen bonds with the oxoanion are Thr124, Thr9 and Ser11 (the last two residues from the TTTS motif, *G. sulfurreducens* numbering). In addition, another conserved and positively charged Arg118 is highly conserved not only in the *Da*G20 TupA, but also in TupA from different *Desulfovibrio* species. This residue is proposed as the structural element conferring the high selectivity of the TupA proteins (Figure 1).

### 2.3. Metal Binding Assays

**Figure 4:**
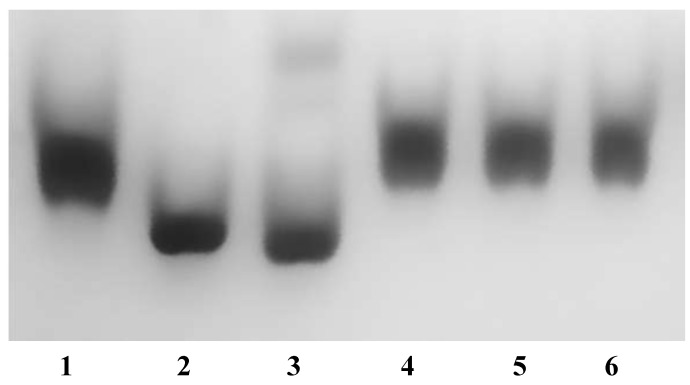
Ligand-dependent mobility shift assays for TupA protein (14 µM) in the presence of different oxoanions (10-fold excess). Lane **1**: TupA; Lane **2**: TupA + MoO_4_^2−^; Lane **3**: TupA + WO_4_^2−^; Lane **4**: TupA + SO_4_^2−^; Lane **5**: TupA + PO_4_^3−^; Lane **6**: TupA + ClO_4_^−^.

Sequence analysis suggests that *Da*G20 TupA is a tungstate-binding protein that is able to bind tungstate and molybdate ions. To test the affinity and specificity of TupA to different anions, native polyacrylamide gel electrophoresis of samples pre-incubated with different oxoanions (MoO_4_^2−^, WO_4_^2−^, SO_4_^2−^, PO_4_^3−^ and ClO_4_^−^) was carried out similar to that described in [8]. The samples were submitted to a gel filtration column prior to loading on native polyacrylamide gel in order to separate the unbound ions and to ensure that differences in mobility were only due to the binding of anions to the protein. As seen in Figure 4, TupA showed a significant mobility shift upon binding to tungstate and molybdate, but not with the other anions. Both molybdate and tungstate induced similar shifts in the mobility of TupA, and incubation with higher concentrations of anions (100-fold) had no visual impact. Quantitative studies of molybdate and tungstate binding were then performed using isothermal titration calorimetry (ITC).

### 2.4. Isothermal Titration Calorimetry (ITC)

ITC has been proven to be a sensitive method to determine affinity constants for tungstate- and molybdate-binding proteins, TupA and ModA, in the nanomolar and subnanomolar ranges [10,12,16]. It has the advantage that nearly all interactions give rise to a heat change, which can be monitored with a high-sensitivity calorimeter, and the binding enthalpy (Δ*H*_obs_) and dissociation constant can be derived. The observed behavior of TupA is consistent with an exothermic process at this temperature (30 °C), with a single binding site model of binding. However the high nature of these bindings precluded an accurate fit to determine the *K*_D_ values. Displacement titrations were done to obtain the correct affinities. The *K*_D_ value of a displacement titration in combination with the *K*_D_ value for the inhibiting ligand in the absence of a strong binding ligand can be used to calculate the actual *K*_D_ for the strong binding ligand (Equation (1; see Section 3.6).

**Figure 5:**
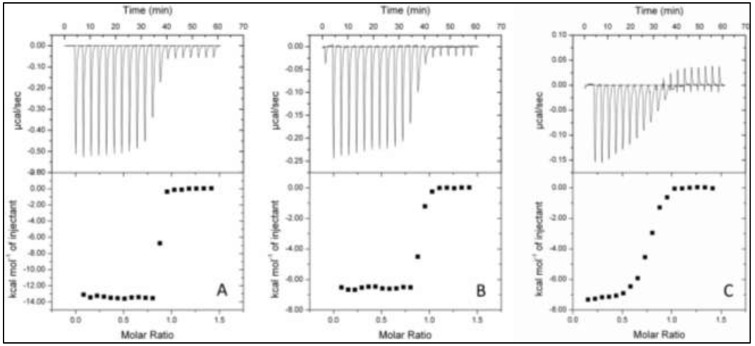
Isothermal titration calorimetry of ligand binding to TupA. TupA (10 µM) was titrated with injections of 100 µM tungstate (**A**) and 100 µM molybdate (**B**); (**C**) Displacement titration of 10 µM TupA incubated with 0.5 nM molybdate, with injections of 100 µM tungstate. Data were fitted with ORIGIN software. The raw ITC data are shown in the top graphs.

ITC of TupA showed that the protein exothermically binds tungstate and molybdate with a stoichiometry of one mole oxoanion per mole of protein, as deduced from the heat release upon the addition of tungstate or molybdate to the protein solution (Figure 5B). Direct titration of sodium molybdate against TupA produced an exothermic binding isotherm with a *K*_D_ value of 6.1 ± 0.9 nM. The value of Δ*H*_obs_ (approximately 6.6 kcal/mol of injectant) is also significantly less favorable, when compared with the tungstate binding. In contrast, the binding of tungstate to TupA is much more exothermic (Figure 5A; Table 1), with Δ*H*_obs_ being increased to approximately 14 kcal/mol of injectant (Table 1). The extremely high affinity of the protein for tungstate resulted in a very steep binding curve, which hampers the determination of *K*_D_. In order to overcome this problem and to determine a *K*_D_ value for tungstate, a binding competition strategy was adopted. A displacement titration of the molybdate-saturated protein with tungstate clearly showed that the protein favors the binding of tungstate, even when the binding site is occupied with a molybdate molecule. The apparent binding constant depends on the concentration of free molybdate, which was 0.5 mM, and therefore, *K*_D_ for tungstate when the protein is saturated with molybdate was determined to be 6.30 ± 0.02 pM (Figure 5C, Table 1). The displacement titration and the extremely low *K*_D_ value for tungstate indicate that the latter should be the physiological substrate for TupA, as expected. The results obtained are in good agreement with those obtained for tungstate binding proteins from *Campylobacter jejuni* [12] and *P. furiosus* [10] and is approximately 1000 times higher than the *K*_D_ value obtained for the *E. acidaminophilum* TupA [11].

**Table 1:** Data for the ITC analysis of oxoanion binding to TupA and ModA proteins at 30 °C.

Protein (+Oxyanion)	Ligand	*n*	*K*_A_ (M^−1^)	*K*_D_ (nM)	∆*H* (kcal·mol^−1^)
TupA	WO_4_^2−^	0.842 ± 0.001	2 × 10^9^ ± 2 × 10^9^	0.5 ± 0.4	−13.500 ± 0.005
	MoO_4_^2−^	0.868 ± 0.002	16 × 10^7^ ± 2 × 10^7^	6.1 ± 0.9	−6.600 ± 0.003
TupA + 0.5 mM MoO_4_^2−^	WO_4_^2−^	0.845 ± 0.003	1600 × 10^8^ ± 6 × 10^8^	6.30 × 10^−3^ ± 0.02 × 10^−3^	−14.60 ± 0.04
TupA + 0.5 mM WO_4_^2−^	MoO_4_^2−^	No displacement			

In each case 10 mM protein was used for the titrations. *n* = measured stoichiometry of binding.

### 2.5. Crystallization and Data Processing

**Figure 6:**
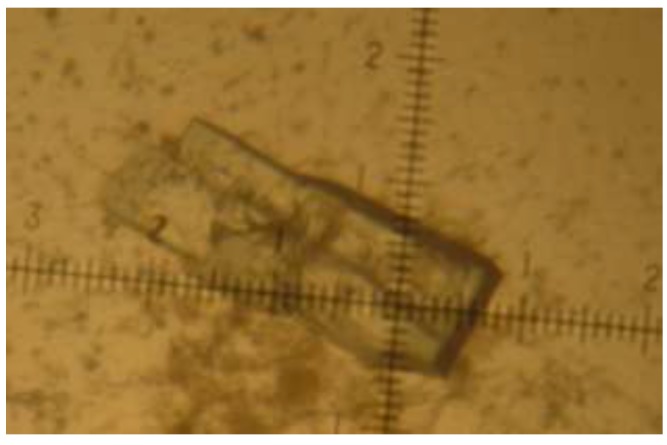
TupA crystal grown in 0.2 M magnesium chloride, 0.1 M HEPES pH 7.5 and 30% (*w*/*v*) polyethylene glycol 3350 solution. Each unit in the scale bar corresponds to 0.1 mm.

To crystallize TupA from *Da*G20, several commercial screens were tested in a 96-well plate using the sitting drop/vapor diffusion method. Plate shaped crystals appeared four days after crystallization setup when using a solution of 0.2 M magnesium chloride, 0.1 M HEPES (4-(2-hydroxyethyl)piperazine-1-ethanesulfonic acid) pH 7.5 and 30% (*w*/*v*) polyethylene glycol 3350 as the precipitating agent (Figure 6).

The scale-up optimization was achieved by varying the protein:precipitant proportion in the crystallization drop, and crystals diffracting up to 1.43 Å resolution were obtained (data collection statistics are presented in Table 2). The crystals belong to the space group P2_1_, and the Matthews coefficient calculation (2.09 Å^3^·Da^−1^) suggests the presence of one molecule of TupA per asymmetric unit and a solvent content of 40.84%. The L test for twinning indicates that these correspond to untwined crystals [31].

**Table 2:** Data collection and processing statistics for the TupA crystal. Values in parentheses correspond to the highest resolution shell.

Data Collection Parameters	
X-ray source	ID23-1 (ESRF, Grenoble)
Detector	PILATUS 6M-F
Wavelength (Å)	0.954
**Processing Statistics**	
Unit-cell parameters (Å, °)	*a* = 52.25; *b* = 42.50; *c* = 54.71; β = 95.43
Space group	P12_1_1
Molecules per AU	1
Matthews coefficient (Å^3^, Da)	2.09
Mosaicity (°)	0.22
Resolution range (Å)	42.50–1.43 (1.45–1.43)
<*I*/σ*I*>	10.3 (2.1)
*R*_merge_ (%) *	4.1 (33.5)
*R*_pim_ (%) ^+^	2.7 (23.4)
R_meas_ (%) ^§^	5.0 (4.1)
Multiplicity	3.0 (2.8)
No. of observed reflections	132,115 (6040)
No. of unique reflections	43,950 (2151)
Completeness (%)	99.1 (98.8)

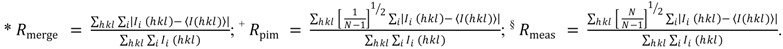

### 2.6. Structure Determination

To solve the structure of TupA, sequence alignments were performed in order to find the best homologous models that could lead to good initial phases obtained by molecular replacement (MR). The available structures deposited in the PDB, from the three families of transporters ModA, WtpA and TupA, have low sequence identity, but a high degree of three-dimensional homology, with very few structural differences. Structure determination was performed with PHASER [32] using as molecular models: a conserved functionally unknown protein from *Vibrio parahaemolyticus* RIMD 2210633 (PBD code 3MUQ) and the *Gs* TupA (PDB code 3LR1). In the first attempts to solve the phase problem, the two homology models were superposed and the non-conserved amino acids were pruned in order to facilitate the rotational and translational searches. Nevertheless, an MR solution could only be obtained when searching for small sections of the protein separately: Section I, from Residues 1 to 81; Section II from Residues 82 to 188; and finally, Section III from Residues 189 to 236. This procedure is commonly used for large, multi-domain or oligomeric proteins, where a high degree of flexibility is expected between the different domains/subunits. In the present case, it suggests that *Da*G20 TupA is also a flexible protein that can adopt multiple conformations. The protein crystal structure is currently under refinement, and the details of the putative tungstate/molybdate binding site are going to be inferred.

## 3. Experimental Section

### 3.1. Bacterial Strains and Plasmids

The *Da*G20 cells were grown in 100-mL rubber-stropped flasks containing 90 mL of medium C from Postgate [33] at 37 °C under anaerobic conditions. The media preparation includes oxygen removal by boiling and bubbling with pure argon for 30 min and sterilization at 121 °C at 20 psi for 20 min. The information on the bacteria strain, plasmid and primers used in this study are given in detail in Table 3.

**Table 3:** Bacterial strains and plasmids used in this study.

Strain/Plasmid/Primer	Properties/Sequence	Source/Reference
*Da*G20	Spontaneously nalidixic acid resistant derivative of G100A, isolated from the production fluids of offshore oil fields in Alaska	Feio, M.J. [21], Hauser, L.J. [34] and Wall, J.D. [35].
pET-46 Ek/LIC vector	*E. coli* cloning vector plasmid	Novagen
NovaBlue GigaSingles cells	*endA1 hsdR17* (rK12^−^ mK12^+^) *supE44 thi-1 recA1 gyrA96 relA1* *lac* [F'p*roA^+^B^+^ lacI^q^ZΔM15*::Tn*10*(Tc^R^)]	Novagen
*E coli* BL21(DE3)	*F*^−^ *ompT gal dcm lon hsdSB*(*rB*^−^ *mB*^−^) *λ*(*DE3* [*lacI lacUV5-T7 gene 1 ind1 sam7 nin5*])	Studier, F.W. [36]
TupA_LIC_Fwd (sense)	GACGACGACAAGATGCTGGAAGTTCTGTTCCAGGGGCCCGAAGCACCGGTTCTTATG	This work
TupA_LIC_Rev (antisense)	GAGGAGAAGCCCGGTTATTCGGCGTTGGGGGT	This work

### 3.2. Cloning of tupA Gene and Protein Expression Optimization

The *tupA* gene (locus tag Dde_0234) was amplified from *Da*G20 cells using the primers included in Table 3. DNA template was obtained from *Da*G20 cells grown until the stationary phase. Briefly, 1 mL of the cell culture was centrifuged, and the pellet was resuspended in 30 µL of sterile deionized water. This suspension was boiled for 5 min in a boiling water bath and then centrifuged at 14,000 rpm for 2 min. A volume of 2 µL of the supernatant was used as the DNA template. The amplification reaction was carried out using FideliTaq™ DNA polymerase (Expand High Fidelity PCR System, Roche, Manneheim, Germany), following the manufacturer’s instructions. The PCR program was as follows: initial denaturation step for 2 min at 92 °C followed by 25 cycles of 92 °C for 30 s, 55 °C for 30 s and 68 °C for 1 min and final extension of 68 °C for 5 min. The amplicon (approximately 800 bp) was purified using the QIAquick extraction kit (Qiagen, Venlo, Netherlands) and quantified by the UV-visible spectrum. The insert (240 ng) was cloned in the pET-46 Ek-LIC vector using the LIC cloning system (Novagen), following the manufacturer’s instructions. NovaBlue GigaSingles competent cells (Novagen) were transformed with the pET46-tupA expression vector, and the plasmid was isolated from a single colony using the NZY-Tech Miniprep kit (NZY-Tech, Lisbon, Portugal). The recombinant plasmids were sequenced using an ABI3700 DNA analyzer (Perkin/Elmer/Applied Biosystems, Stabvida, Caparica, Portugal). The sequences were analyzed and aligned using the online tool, BLASTp [37], and ClustalW [38].

BL21 (DE3) cells were transformed with the pET46-tupA expression vector, and the protein production was evaluated at different concentrations of IPTG (0, 0.2, 0.5 and 1.0 mM) and the induction time (3 h, 5 h and overnight). To test whether TupA is produced as a soluble protein, the BugBuster reagent (Novagen) was used as per the protocol.

### 3.3. Protein Expression and Purification

*E. coli* BL21 (DE3) cells containing the pET46-tupA were cultured in sterile Luria-Bertani medium containing ampicillin (100 µg/mL) at 220 rpm and 37 °C. When the OD_600_ reached 0.4 AU, cells were induced with 0.1 mM IPTG during 3 h at room temperature. The cells were collected by centrifugation at 7000 rpm for 15 min, washed in 5 mM Tris-HCl buffer, centrifuged at 7000 rpm for 15 min and resuspended again in 5 mM Tris-HCl buffer containing DNase (5 µg/mL) at a ratio of 2 g cells/mL. The cell suspension was freeze and thawed thrice before disrupting the cells on a French press cell at 150 psi. The crude extract was centrifuged at 9000 rpm for 30 min, ultracentrifuged using a Beckman Coulter Optima™ LE-80K ultracentrifuge (Beckman Coulter, Inc., Fullerton, CA, USA) at 45,000× *g* for 45 min and the soluble fraction was filtered through a 0.45 µm membrane. Although the pET-46 Ek/LIC expression vector encoded a six-histidine tag at the *N*-terminal sequence, attempts to purify TupA using immobilized-metal affinity chromatography (IMAC) failed to bind the protein to the resin. Hence, the strategy to purify TupA was changed to the protocol described as follows. The first purification step involved the loading of the soluble extract into a DEAE Sepharose Fast Flow (GE Healthcare Bio-Sciences AB, Uppsala, Sweden) resin equilibrated with 3 column volumes (CV) of 5 mM Tris-HCl (equilibration buffer). After protein loading, the resin was washed with equilibration buffer to remove the unbound proteins, and TupA was eluted using a gradient from 5 to 500 mM Tris-HCl buffer in 8 CV. The protein fractions collected were analyzed by 12% SDS-PAGE stained using Coomassie blue. The fractions containing TupA were concentrated and loaded onto a Superdex 75 HR10/300 GL column (GE Healthcare Bio-Sciences AB, Uppsala, Sweden) equilibrated with 50 mM potassium phosphate buffer containing 150 mM NaCl. The fraction containing the pure protein was pooled, concentrated and stored at −80 °C until further use. All of the steps, including cell collection, soluble extract preparation and the purification procedure, were performed at 4 °C and pH 7.6.

### 3.4. Extinction Coefficient Determination

The extinction coefficient was determined by measuring the absorbance at 280 nm of a pure TupA protein sample quantified using the Bradford method [39] with bovine serum albumin as the standard. The UV-visible absorption spectrum was performed on a Shimadzu UV-2101PC split beam spectrophotometer (Shimadzu, Shimadzu, Japan) using 1-cm optical path quarts cells. The value obtained was in agreement with the one determined using the bioinformatic tool, ProtParam, from the ExPASy portal [29].

### 3.5. Protein Gel Shift Assay

TupA gel shift assays were performed following the protocol described by Rech *et al.* [8]. Briefly, TupA protein samples (14 µM) were incubated with MoO_4_^2−^, WO_4_^2−^, SO_4_^2−^, PO_4_^3−^ and ClO_4_^−^ anions (140 µM) in 25 mM Tris-HCl (pH 7.5) buffer at room temperature for 25 min. Unbound anions were separated from TupA with a PD10 desalting column (GE Healthcare Bio-Sciences AB, Uppsala, Sweden). Protein samples were mixed with 0.25 volume of sucrose solution (30% *w*/*v*) containing bromophenol blue and resolved on a native 12% polyacrylamide gel buffered with 50 mM Tris-HCl (pH 8.5). The electrophoresis was carried out at 100 V, 100 A and 4 °C using a 0.1 M Tris-HCl and 0.1 M glycine (pH 8.3) running buffer. The mobility shift assay after anion binding was visualized through the staining of the gel with Coomassie Blue staining solution.

### 3.6. Isothermal Titration Calorimetry

Isothermal titration calorimetry experiments were performed using a VP-ITC calorimeter (MicroCal Inc., GE Healthcare, Pittsbugh, PA, USA). Prior to experiments, protein was dialyzed extensively against the reaction buffer (5 mM Tris-HCl (pH 7.5)) made with ultrapure water (Milli Q system, Millipore AB, Sweden). Binding protein (10 µM) was equilibrated in reaction buffer at 30 °C in the cell of the calorimeter, and subsequently, 20 or 23 injections of 10 µL of a 100 µM sodium tungstate or molybdate solution were performed and the heat response recorded. After subtraction of the baseline, the integrated heat responses were fitted to the single binding site model using the ORIGIN software package (Northampton, MA, USA) supplied with the calorimeter. For competition experiments, the reaction buffer was supplemented with the stated concentrations of molybdate prior to the injections with sodium, tungstate or the reverse. The relationship between apparent binding affinity of the high-affinity ligand (*K*_app_) and the underlying constants is derived from Equation (1) [40]:

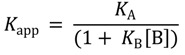
(1)
where *K*_A_ is the binding constant for the strong binding ligand and *K*_B_ is that for the competitively inhibiting ligand. The apparent binding constant depends on the concentration of the free competitively inhibiting ligand (B) [40].

### 3.7. Crystallization

TupA protein was concentrated up to 7.5 mg/mL in 5 mM Tris-HCl (pH 7.5) with a Vivaspin 20 ultrafiltration device (Sartorius Stedim Biotech S.A., Goettingen, Germany). The final concentration of TupA was determined from the absorbance at 280 nm, using an extinction coefficient of 30,440 M^−1^·cm^−1^.

The first crystallization trials were performed at 20 °C using the sitting-drop vapor diffusion method, with 0.5 µL of protein: 0.5 µL of precipitant solution on 96-well crystallization plates (SWISSCI 'MRC' 2-Drop Crystallization Plates, Douglas Instruments, Berkshire, UK). Several commercial screens were used, namely the PEG/Ion HT (Hampton Research, Aliso Viejo, CA, USA), the JBScreen Classic 1-10 (Jena Bioscience, Jena, Germany) and an 80 conditions in-house screen (based on the screen of Jancarik *et al.* [41]). The TupA crystallized in only one of the conditions of the in-house screen containing 0.2 M magnesium chloride, 0.1 M HEPES (pH 7.5) and 30% (*w*/*v*) polyethylene glycol 3350. Colorless plate-shaped crystals appeared within 4 days (Figure 6).

Scale-up and optimization experiments were performed, and new crystals with maximum dimensions of 0.3 × 0.15 × 0.06 mm^3^ appeared in hanging-drops with 2 µL of protein (at 7.5 mg/mL): 1 µL of precipitant solution on a 24-well crystallization plate. These crystals were used to for data collection.

### 3.8. Data Collection and Processing

The crystals were flash-cooled directly in liquid nitrogen, using Paratone as the cryoprotectant, and maintained under a stream of nitrogen gas during data collection.

A complete dataset was collected at beamline ID23-1 at the European Synchrotron Radiation Facility (ESRF, Grenoble, France) and the crystal diffracted up to 1.43 Å at a wavelength of 0.954 Å. The TupA crystal belongs to the monoclinic space group, P2_1_, with the unit-cell parameters: *a* = 52.25 Å, *b* = 42.50 Å, *c* = 54.71 Å and β = 95.43°. The Matthews coefficient was calculated (*ca.* 2.09 Å^3^/Da) [42], suggesting the presence of one monomer (α) per asymmetric unit, with a solvent content of 40.84%.

Data was processed with the XDS package [43] and AIMLESS [44] from the CCP4 program package v. 6.3.0 (Collaborative Computational Project, Number 4, 1994) [45]. The data collection and processing statistics are presented in the Table 2.

## 4. Conclusions

The transport of tungstate and other analogous oxoanions, like molybdate, is very relevant in organisms that contain key metabolic W/Mo-enzymes, like *Desulfovibrio* species. Despite this, there are no reports about the characterization of molybdate/tungstate uptake systems from these SRB. An analysis of the *Desulfovibrio* genome annotated to date shows that molybdate and tungstate transporters are encoded in the chromosome of these organisms and belong to the Mod and Tup family of proteins, respectively [1]. Although Mo and W have similar biochemistry [46], molybdate and tungstate transporters can differentiate between them. The molecular basis of the selectivity by the Tup and Mod transporters remains to be understood. Valuable information can be derived from the biochemical and structural characterization of the TupA protein and, particularly, from organisms that contain both (Mod and Tup) kinds of transporters. In this work, we report the expression, purification, preliminary characterization, crystallization and structure determination of *Da*G20 TupA. In order to attest to the binding of molybdate and tungstate to *Da*G20 TupA, gel shift assays were also carried out. Different from the TupA from *Eubacterium acidaminophilum* [9], *Da*G20 TupA not only efficiently binds tungstate, but also molybdate anions. In order to quantitatively determine the binding affinity of TupA towards the two oxoanions, isothermal titration calorimetry was carried out. The obtained data show that TupA binds in a 1:1 stoichiometry the two anions, but has much higher affinity to tungstate than to molybdate (around a 1000-times lower *K*_D_ value for tungstate anions). Furthermore, in a competitive binding assay, the protein is capable of displacing the molybdate in order to bind what we think is its physiological partner, tungstate. In order to understand the specificity of TupA, site-directed mutagenesis is under way, where some of the putative key residues for binding are going to be inspected.

Conditions to crystallize TupA were found, and the crystals diffract up to 1.43 Å. The high resolution structure will allow the detailed characterization of the ligand pocket, coordination geometry and conformational changes upon metal binding, which will help to better understand the mode of action of these transporters.
